# Loewenstein Occupational Therapy Cognitive Assessment to Evaluate People with Addictions

**DOI:** 10.1155/2017/2750328

**Published:** 2017-01-10

**Authors:** Gloria Rojo-Mota, Eduardo J. Pedrero-Pérez, José M. Ruiz-Sánchez de León, Irene León-Frade, Patricia Aldea-Poyo, Marina Alonso-Rodríguez, Jara Pedrero-Aguilar, Sara Morales-Alonso

**Affiliations:** ^1^Institute of Addictions, Madrid Salud, Madrid City Council, Madrid, Spain; ^2^King Juan Carlos University, Madrid, Spain; ^3^Complutense University of Madrid, Madrid, Spain

## Abstract

**Background:**

The LOTCA (Loewenstein Occupational Therapy Cognitive Assessment) battery is a cognitive screening test which is widely used in occupational health. However, no work has been found that explores its use in addiction treatment.

**Objectives of Study:**

To explore the convergent validity of LOTCA with neuropsychological tests that assess related cerebral functional areas.

**Methods:**

The LOTCA, along with a battery of neuropsychological tests, was administered to a sample of 48 subjects who start a treatment by substance or gambling addictions.

**Findings:**

A correlational pattern was observed of a considerable magnitude between the effects of the LOTCA scales and those of some neuropsychological tests, but not with others. There is barely any convergence in measures with memory and executive function tests.

**Relevance to Clinical Practice:**

There is a lack of research applying test of occupational assessment to populations of patients treated by addictive behaviors. The LOTCA seems to be a reliable and valid test for preliminary screening of function in certain cognitive areas, easy, and quick to use (around 30 minutes). However, it must be supplemented with other tests for a full and ecological assessment of patients.

**Limitations:**

An incident, small-size sample.

**Recommendations for Further Research:**

New studies are needed to explore the applicability, diagnostic validity, and whole psychometric quality of the test in addiction-related treatment.

## 1. Introduction

Substance addiction is a health problem that affects all areas of individual functioning and their relation with environment. There are many ways to view addiction: from considering it a brain disease [1] to understanding it as a behavior more or less effective to cope with difficulties in life, which can be reversed even spontaneously without requiring medical treatment [2, 3]. In any case, is no doubt that “addictive behavior occurs within a social context, which can serve as a risk or protective factor as social contexts and individuals influence one another” ([4, p. 353]). Contrary to prior thinking, it has been recently suggested that addiction does not merely disrupt a person's occupational life; but rather, it constitutes a person's occupational life [5]. The establishment of habits and routines related to addiction impregnates a person's life in such way that only that which is related with drug use seems significant to them. The therapist's work lies in providing real life activities in a natural environment which have become dysfunctional throughout the addiction, abandoned, interrupted, or not accessed.

However, there is one significant difficulty in designing individual occupational treatments: the cognitive deficits which are often associated with the addiction. This happens in both cases of substance addictions [6] and cases of behavioral addictions [7]. Such deficits can represent preaddiction conditions and, therefore, vulnerabilities to developing the addiction, or effects deriving from the consumption of substances due to either the specific biochemical effects or the associated stress or restriction of stimulation or even a combination of both [8]. A comprehensive review found that certain cognitive deficits represent common addictive processes elements (deficits in episodic memory, emotional processing, and the executive components of updating and decision-making), while others are specifically associated with each substance (psychostimulants and alcohol use with impulsive action and cognitive flexibility, alcohol and MDMA with spatial processing, perceptual speed and selective attention, cannabis and methamphetamine on prospective memory deficits, and cannabis and MDMA on processing speed and complex planning) [9]. A change in habits involves cognitive and metacognitive skills that often reflect deficient performance in people with addictions [10], which is related to a “shut-down” of the prefrontal cortex, which stops exercising control over other cerebral areas [11]. Previous studies have shown that neuropsychological alterations are associated with reduced level of involvement and participation of drug dependent individuals in treatment programs and with a higher rate of dropping out of these programs [12], to the extent that individuals with addictive behaviors may have considerable difficulty in becoming aware of their own deficit, to understand and reason complex instructions, to inhibit impulsive responses, to plan their daily activities, and to make everyday decisions [13]. Their detection by cognitive assessment allows designing rehabilitation programs that are aimed to better use of other therapeutic resources available, such as cognitive therapy or therapy relapse prevention [14].

## 2. Literature Review

According to a recent review, occupational therapists have been participating in teams treating people with addictions for decades although their scientific production is quite scarce [15]. This review found a critical deficit in the availability of occupational assessment instruments, which have been validated in the field of addictions. Currently, they are only available nonreplicated works on the Executive Function Performance Test (EFPT) [16], the Assessment of Motor and Process Skills (AMPS) [17], the Allen Cognitive Level Screen-5 (ACLS-5) [18], and little more. The review concludes the need to validate occupational instruments in people with addictions who are undergoing treatment as an urgent need in order to promote and improve the scientific production of the profession in this clinical field.

One of the most widely used instruments in occupational health to assess cognitive performance is the Loewenstein Occupational Therapy Cognitive Assessment (LOTCA) battery [19]. It provides an assessment of basic cognitive skills required for everyday function, including orientation, visual perceptual and psychomotor abilities, problem-solving skills, and thinking operations. The results can also be used in treatment planning and to review progress over time. The test has been used in a large number of studies and its psychometric properties have proven satisfactory in many research projects and in very diverse populations and geographic areas. Just to mention a few of the most recent, it has been used in the geriatric population [20], for brain damage [21] and cases of schizophrenia [22], among others. It has also been used as a tool for measuring the effectiveness of treatments of stroke patients [23–26], in healthy individuals [27] and schoolchildren [28], among many other people [29]. No work has been found which uses LOTCA in a population of people with substance addiction undergoing treatment.

## 3. Study Objectives

The objective of this work is to apply LOTCA to a sample of people receiving treatment for substance addiction and to explore its convergent validity with other occupational and neuropsychological tests used to estimate cognitive deficits.

## 4. Methods

### 4.1. Participants and Procedures

The sample was obtained from a specific urban, public, and free center that treats people with substance addictions in the city of Madrid: the San Blas CAD Drug Addiction Care Center (Addiction Institute, Madrid Health, City of Madrid). The people access this service directly and are assessed using diagnostic interviews by a multidisciplinary team (doctors, nurses, psychologists, social workers, and occupational therapists). The criterion for inclusion in this study was complying with the requirements for being diagnosed with a Substance-Related Disorder, as per the DSM-5 [30]. The criteria for exclusion were having previously suffered any type of brain damage (*n* = 0), presenting some type of psychotic process in an active phase at the time of the initial assessment (*n* = 0), or presenting any condition that would prevent the proper comprehension of the instructions used to complete the tests such as language issues, illiteracy, and a state of confusion (*n* = 1). The time necessary in each case (generally between one and two weeks) for participants to be no longer under the effect of any nonprescribed substances was allowed to pass before the assessment. When necessary, this was confirmed with the visible ultraviolet spectrophotometry method to find metabolites of opiates, cocaine, cannabis, and benzodiazepines in the urine or breath alcohol testing (one subject was excluded due to active consumption upon assessment). The participants were informed of the two objectives of the assessment: to design an individualized treatment and to do research work. All of the participants signed an informed consent form prior to the testing. The study was authorized by the Research Unit of the Madrid Health Evaluation and Quality Department and was approved by the Addiction Institute Research Committee.

In order to do this study, the first 50 subjects beginning treatment with the service were recruited. The occupational tests were administered simultaneously by two occupational therapists (principal and observant evaluator) and the neuropsychological tests by three neuropsychologists (each patient was assigned to one of them). All of the participants underwent occupational testing and the battery of neuropsychology tests in a period of less than one week. Two of them did not finish the assessment because they did not want to, which means that the final sample was *n* = 48 subjects. The sample descriptors can be found in Table 1.

### 4.2. Instruments

The Loewenstein Occupational Therapy Cognitive Assessment (LOTCA) battery, which is a performance test, contains 20 items categorized into four subscales: orientation (2 items), perception (6 items), visuomotor organization (7 items), and thinking operations (5 items; the score for the pictorial sequence is obtained with the average of two tasks, A and B). The LOTCA kit contains testing materials (card decks, coloured blocks, pegboard set, and other materials) and a manual that includes definitions of the cognitive domains assessed, instructions for administration, and specific scoring guidelines. The scores were placed on a Likert-type scale ranging from 1 to 4 (with the exception of items 1–5 regarding categorization, unstructured Risk Object Classification [31], and structured ROC). The higher the scores were, the better the performance was. Because it is not meaningful to add up unrelated areas of cognitive and perceptual skills, we did not calculate a total score for the LOTCA. The time each participant took to complete the LOTCA was recorded as a proxy of the participant's information-processing speed. The LOTCA was administered and scored using the instructions in the test manual.

The Allen Cognitive Level Screen-5 (ACLS-5) [32] consists of a piece of leather perforated on all sides, leather laces, two types of needle, and a shoelace. Administration time is approximately 10 minutes. The assessment allows the clinician to evaluate the person's performance on three different leather-lacing tasks of varying challenge. The test consists of three visual motor learning tasks (stitching leather) with increased complexity in the activities. In order to complete the three tasks successfully, the person must pay attention to the verbal instructions and demonstration, understand and use sensory signals from the materials (leather, lace, and needles), and effectively use feedback from the motor actions during practice with the lacing materials. The test was applied with satisfactory results on subjects being treated for substance addiction and evidence was found of convergent validity with neuropsychology tests [32].

The battery of neuropsychology tests was based on prior suggestions for cognitive assessment among populations of people being treated for addiction [33]. Specifically, the following were administered in accordance with the standards found in each manual: the Brief Cognitive Status Exam (BCSE) [34] as a measure of one's overall mental state, the Wechsler Adult Intelligence Coding and Symbol Search to establish the Processing Speed Index (PSI), Digit Span and Symbol Span of the Wechsler Memory Scale [35] to assess working memory, the Rey-Osterrieth Complex Figure Test [36], and the Logic Memory Test to assess visual memory and verbal memory, as well as the Five-Digit Test [37], an alternative to the Stroop test, to assess inhibition and flexibility.

### 4.3. Data Analysis

The internal consistency of the test was studied using Cronbach's *α*. A confirmatory factor analysis was done on the theoretical structure of the survey using the unweighted least squares method since ordinal categorical items were used and normality was not expected in the distribution of the data. The indicators provided by the AMOS 18 program were used to study the adequacy of the theoretical model to the data: RMR = Root Mean Square Residual, with acceptable values below 0.06; GFI = General Fit Index; AGFI = Adjusted Goodness of Fit Index; NFI = Normed Fit Index; RFI = Relative Fit Index, all of which showed acceptable values above 0.90; PNFI = Parsimonious Normed Fit Index; PGFI = Parsimony Goodness of Fit Index, both acceptable with values above 0.7. Spearman's rho was used to study the correlations [38]. The comparisons between subgroups were made using nonparametric procedures. The statistical package SPSS 19 was used for these analyses.

## 5. Findings

Table 2 outlines the descriptors of the items obtained upon application to the sample. On the whole, the test showed reliability of Cronbach *α* = 0.854. The confirmatory factor analysis showed suitable indicators of adequacy for the four-dimension theoretical model for the sample data (RMR = 0.05; GFI = 0.96; AGFI = 0.95; NFI = 0.94; RFI = 0.93; PNFI = 0.86; PGFI = 0.75).

Table 3 shows the correlations found between the LOTCA scales and the neuropsychology test scales. There were no significant differences between the scores obtained by men and women on the LOTCA scales (0.44 < *Z* < 1.71; *p* > 0.05 in all cases), or the primary drug (0.92 < *χ*^2^_(d.f.  3)_ < 4.01; *p* > 0.05 in all cases). However, the difference in the time used by men and women to complete the test was significant: the men used less time (mean = 24.32; SD = 13.12) than the women (mean = 34.90; SD = 10.55). There were significant differences in relation to the education level of the participants: the higher the scores, the higher the education level on all scales (7.69 < *χ*^2^_(d.f.  3)_ < 12.42; *p* > 0.05 in all cases), except orientation (*χ*^2^_(d.f.  3)_ = 1.84; *p* = 0.61), and in the time used to complete the test (*χ*^2^_(d.f.  3)_ = 3.12; *p* = 0.37).

## 6. Discussion

LOTCA is a relatively systematic test that can be useful for initial assessments of people who are beginning treatment for addictive behaviors with or without substances. However, its use among this clinical population has never before been established. As a screening test of function areas, the results must show correlation with more demanding and systematic tests used in the field of neuropsychology. This work explored the existence of a relationship between both types of tests. The results are conclusive: there are correlations of a considerable size of effect with some tests, but not with others. Specifically, LOTCA does not seem to measure something related to executive functions given that it barely shows convergence in measurements with the 5-Digit Test.

However, there are correlations of a great effect size with other screening tests both in the field of occupational therapy (ACLS-5) and in the field of neuropsychology (BSCE). Specifically, the ACLS-5 is a motor test which includes comprehension of instructions, executive planning, and the ability to resolve conflicts that arise during its performance. These three abilities are measured by LOTCA and reflect considerable measurement convergence. The same occurs with the BCSE, which also explores these abilities, among others, although it is more aimed at the establishment of clinical cut-off points than differentiated estimation of the various abilities. To this end, LOTCA seems to be a good cognitive screening instrument.

There are also significant correlations with those obtained on the Rey Figure. This test, which is in the copy phase, involves visuomotor skills used to detect stimulus and their copying. In a second phase, it requires recent memory and maintaining copied sketches in the memory in addition to the motor skills.

There are also significant correlations with attention span tests as concerns both numbers and other types of symbols. However, the correlations shown are barely noticeable (and the fact that they appear due to a random effect cannot be discarded) when memory or executive function tests are involved.

The LOTCA orientation scale barely correlates with neuropsychology tests. This scale is likely useful in more serious cerebral function pathologies but not in addiction subjects, whose cognitive deficits are minor and do not affect such basic functions. Proof of this lies in the mean obtained by the sample studied being very close to the maximum possible (7.73 out of 8) which means that it is not related to the performance level in the specific areas studied by other scales.

## 7. Limitations

This work has limitations that should be considered. The main limitation is the sample size, which is not sufficient enough to absolutely affirm the relationships observed. The method for obtaining the sample is merely incidental as the first subjects to arrive at the center's occupational therapy service were recruited which means that it is not possible to generalize the results obtained. Finally, there are newer versions of LOTCA than the one used which could provide all-new data not contemplated by this initial version.

## 8. Recommendations for Further Research

The nonexistence of research work where this test is applied to people with addictions means that this is a preliminary study that must be replicated on larger samples and with better sample selection methods. In conclusion, LOTCA is a cognitive screening test that allows for an approximation of some areas of cognitive function yet not all meaning that it must be supplemented with other tests that measure executive function and memory. As a screening test, it may be used ahead of others that are widely used in occupational therapy (i.e., ACLS-5) and neuropsychology (i.e., MMSE, MoCA, and BCSE) to the extent that, beyond estimating a general cut-off point of good or poor function, it explores the quality of execution in specific functional areas. In any case, this test does not make it possible to conduct a full neuropsychological assessment but rather simply quickly detects when and who should undergo it and in which areas more important deficits are detected. To this end, its use can encourage an understanding by a therapist of the more deficient areas of function, to refer patients to other professionals as necessary to conduct more in-depth assessments, to design intervention plans considering a patient's most important deficits, and to estimate the change generated after occupational intervention [39]. To this extent and with the precautions indicated in mind, its inclusion in the battery of tests administered by therapists participating in the treatment of people with addictions is recommended. New studies are needed to contribute more information on its potential and on the rest of the tests which must be used to supplement its use in order to provide a full occupational assessment that is adequate for people in rehabilitation processes for their addictive behaviors.

## Figures and Tables

**Table 1:** Descriptives of the sample.

	Gender		
	Male	Female	Total

*n*	38	10	48
%	79.2	20.8	100

	Age		
Mean	37.5	48.5	39.8
SD	12.2	10.7	12.6
Rank	18–62	32–67	18–67

	Academic level (%)		
Primary or less	23.4	30.0	25.0
Secondary (obligatory)	36.8	20.0	33.3
Secondary (advanced)	36.8	30.0	35.4
Universitary	2.6	20.0	6.3

	Main drug (%)		
Heroine	5.3	—	4.2
Cocaine	44.7	30.0	41.7
Alcohol	34.2	70.0	41.7
Cannabis	15.8	—	12.5

**Table 2:** Scores obtained on the LOTCA scales and items.

	Score	Mean	Minimum	Maximum	SD
Orientation	2–8	7.73	6	8	0.50
Time	1–4	3.91	2	4	0.36
Place	1–4	3.82	3	4	0.39
Perception	6–24	22.52	18	24	2.09
Object identification	1–4	4.00	4	4	0.00
Shape identification	1–4	3.73	3	4	0.45
Overlapping figures	1–4	4.00	4	4	0.00
Object constancy	1–4	3.98	3	4	0.15
Spatial perception	1–4	3.91	2	4	0.36
Praxis	1–4	3.88	1	4	0.55
Visuomotor organization	7–28	24.18	15	28	3.59
Copying geometric forms	1–4	3.55	2	4	0.59
Reproducing a two-dimensional model	1–4	3.59	2	4	0.62
Constructing a pegboard design	1–4	3.66	1	4	0.71
Constructing a colored block design	1–4	3.66	1	4	0.81
Constructing a plain block design	1–4	3.20	1	4	1.00
Reproducing a puzzle	1–4	3.05	1	4	0.83
Drawing a clock	1–4	3.48	2	4	0.70
Thinking operations	5–23	18.26	11	23	3.51
Categorization	1–5	3.84	2	5	1.12
ROC: unstructured	1–5	3.64	1	5	1.24
ROC: structured	1–5	3.70	1	5	1.00

Pictorial sequence A	1–4	3.91	3	4	0.29
Pictorial sequence B	1–4	3.48	1	4	0.90

Geometrical sequence	1–4	3.39	1	4	0.97
Length of time (minutes)		29.84	14	55	10.52

**Table 3:** Spearman correlations between the LOTCA scale scores and scale scores obtained with the neuropsychology battery of tests.

	LOTCA				
	Orientation	Visual perceptual abilities	Psychomotor abilities	Thinking operations	Execution time
ACLS-5	0.08	0.34^*∗∗*^	0.53^*∗∗∗*^	0.48^*∗∗∗*^	−0.44^*∗∗∗*^
BCSE	0.26^*∗*^	0.55^*∗∗∗*^	0.54^*∗∗∗*^	0.51^*∗∗∗*^	−0.58^*∗∗∗*^
Rey Complex Figure					
Copy	0.33^*∗*^	0.47^*∗∗∗*^	0.55^*∗∗∗*^	0.43^*∗∗*^	−0.32^*∗*^
Immediate recall	0.11	0.44^*∗∗∗*^	0.56^*∗∗∗*^	0.41^*∗∗*^	−0.31^*∗*^
WAIS-IV					
Coding	0.14	0.51^*∗∗∗*^	0.59^*∗∗∗*^	0.59^*∗∗∗*^	−0.20
Symbol search	0.04	0.46^*∗∗∗*^	0.64^*∗∗∗*^	0.50^*∗∗∗*^	−0.37^*∗∗*^
Processing speed Index	0.10	0.54^*∗∗∗*^	0.68^*∗∗∗*^	0.60^*∗∗∗*^	−0.17
Digit span	0.33^*∗*^	0.68^*∗∗∗*^	0.59^*∗∗∗*^	0.51^*∗∗∗*^	−0.41^*∗∗*^
WMS-IV					
Symbol span	0.28^*∗*^	0.50^*∗∗∗*^	0.52^*∗∗∗*^	0.54^*∗∗∗*^	−0.31^*∗*^
Logic memory I	0.27^*∗*^	0.24	0.30	0.30^*∗*^	−0.26
Logic memory II	0.24	0.27^*∗*^	0.37^*∗∗*^	0.34^*∗*^	−0.30^*∗*^
Five-Digit Test					
Reading	0.20	0.41^*∗∗*^	0.33^*∗*^	0.24	−0.21
Counting	0.15	0.25^*∗*^	0.31^*∗*^	0.16	−0.23
Choosing	0.06	0.35^*∗*^	0.20	0.16	−0.11
Switching	−0.04	0.23	0.25^*∗*^	0.23	−0.29^*∗*^
Inhibition	−0.12	0.21	0.08	0.05	−0.23
Flexibility	−0.12	0.16	0.22	0.20	−0.15

Note: ^*∗∗∗*^*p* < 0.001; ^*∗∗*^*p* < 0.01; ^*∗*^*p* < 0.05.
